# Results of a national survey among occupational physicians to estimate the number of workers with active medical devices and their types in the context of managing electromagnetic hazards

**DOI:** 10.3389/fpubh.2025.1599754

**Published:** 2025-07-28

**Authors:** Rebecca Gasparini, Fabriziomaria Gobba, Andrea Bogi, Giovanni Calcagnini, Federica Censi, Rosaria Falsaperla, Eugenio Mattei, Cecilia Vivarelli, Simona D'Agostino, Alberto Modenese

**Affiliations:** ^1^Department of Biomedical Metabolic and Neural Sciences, University of Modena and Reggio Emilia, Modena, Italy; ^2^Physical Agents Sector, Regional Public Health Laboratory, South-East Tuscany Health Unit, Siena, Italy; ^3^Department of Cardiovascular, Endocrin-metabolic Diseases and Aging, Italian Institute of Health, Rome, Italy; ^4^Department of Occupational and Environmental Medicine, Epidemiology and Hygiene, Italian National Institute for Insurance against Accidents at Work, Rome, Italy; ^5^Department of Information Engineering, Electronics and Telecommunications, Sapienza University of Rome, Rome, Italy

**Keywords:** electromagnetic interference, active implanted medical devices, active wearable medical devices, occupational electromagnetic fields exposure, workers' health surveillance

## Abstract

**Introduction:**

Occupational exposure to electromagnetic fields (EMF) is almost ubiquitous: workers with Active Implantable Medical Devices (AIMD) and Active Wearable Medical Devices (AWMD) are considered at particular risk with respect to occupational risk assessment and health surveillance (HS) procedures, due to possible electromagnetic hazards resulting in interference problems. The identification of these workers is therefore fundamental for prevention purposes. Aim of the study is to provide an estimate of the proportion of workers with AIMD and AWMD, and to list the main types of devices involved.

**Methods:**

We performed an online survey among a sample of Occupational Physicians (OPs) from two Italian Regions.

**Results:**

132 OPs responded, based their answers on a whole number of more than 200,000 workers visited within HS activities in the previous year. Our results show that the 0.8% of the working population in Italy can be estimated as “particularly at risk” for EMF exposure at the workplaces due to the presence of AIMD or AWMD. The most common AIMD resulted pacemakers and implanted cardioverter defibrillators, involving the 0.24% of the workers. Considering AWMD, the most common resulted hearing aids and hormones/drugs pumps, respectively worn by the 0.25 and the 0.17% of the working population.

**Discussion:**

It should be taken into account that potential interference problems could also occur for levels of exposure to EMFs comparable to those measurable for non-professionally exposed populations. Accordingly, the conditions of increased susceptibility to EMF hazards must be carefully considered for a proper occupational EMF risk prevention: the data presented in this work provide a solid foundation for quantifying the actual impact of workers with AIMD and AWMD in the workplaces, and the main types of devices involved.

## 1 Introduction

Exposure to electromagnetic fields (EMF) at the workplaces is nowadays (1) widespread across various sectors, including industry, healthcare, aesthetic, and services. The large majority of the working population is exposed to EMF levels well below the reference levels for occupational exposure recommended by the International Commission on Non-Ionizing Radiation Protection (ICNIRP) (2, 3) and, accordingly, in Europe by the EU Directive 2013/35/EU (4). In many cases, EMF exposure in the workplace is even lower than the ICNIRP reference levels established for the general public. Nevertheless, electro-magnetic interference (EMI) for workers with active implantable medical devices (AIMD) and active wearable medical devices (AWMD) might occur in case of exposure to EMF levels within the permissible occupational levels, and all the frequency bands of the spectrum can be interested. Indeed, cases of electromagnetic interference phenomena in patients with AIMDs or AWMDs have been documented in both everyday life and occupational scenarios, mainly for cardiac devices (5, 6). The EU Directive 2013/35/EU takes these workers into account, defining them as “workers at particular risk” and requiring special attention in the assessment and prevention of the EMF-related risk, as well as in health surveillance (HS) programs (4) for workers exposed to EMF.

A couple of brief necessary notes here are required. The first is related to occupational HS: within this manuscript, the concept of HS is specifically referred to the context of the Italian country, where occupational HS is declined as a medical practice, performed only by trained Occupational Physicians (OPs) with a mandate from the companies. Accordingly, HS is a fundamental preventive measure, i.e. an example of secondary prevention from a medical perspective, and it is mandatorily activated for all the workers identified as exposed to occupational hazards based on the companies' OSH risk assessment documents. A direct consequence of this is that in Italy almost all the workers undergo medical examination from OPs under HS programs. The only exceptions could be workers who are self-employed and do not want to activate this preventive measure for themselves or employees with a merely office work with less than 20 h/week of activities in front of visual display units and no other relevant occupational hazards exposing them, including e.g., job stress or ergonomic hazards (7, 8). OPs are supposed to perform a full medical examination of the workers (e.g. not only checking with an audiometer the hearing function of workers exposed to noise, avoiding a full inspection of other apparatus and an adequate anamnesis) and decide on their fitness for work, based on the hazards they are exposed to. Accordingly, in case of a recognized occupational EMF hazard based on companies' risk assessment documents, but also in case of an absence of this specific hazard, OPs are supposed to identify workers with AIMD or AWMD, with their direct examination and/or with a detailed anamnesis. In case of a potential electromagnetic hazard, OPs need to inform the workers of the possible EMI risk, recommending them to keep an adequate distance from the EMF emitting sources, to inform the medical specialist responsible for the device in case of any suspected problem and, if they believe these problems are related to the job, to ask for further examinations with the OPs (with the possibility that the workers decide to inform also the company if they feel that this may help in finding a more practical and quick solution to the issue, e.g. identifying specific individual preventive measures to avoid any risk).

The second important note is related to occupational EMF exposure. EMF are, undoubtedly, a recognized potential risk factor, and as anticipated almost all the workers may have a certain level of exposure to them, but of course this cannot be considered sufficient to define them as “occupationally exposed”. In principle, specific preventive measures should be provided for the workers whose work-related EMF exposure can increase the risk for their health and/or safety: from this point of view, these are the workers that should be considered “occupationally exposed”, hence deserving the implementation of specific protective measures, including HS. But a clear definition of these workers is really problematic: for the biophysical short term direct and indirect (e.g. contact currents) effects in workers not “at particular risk” the relations with exposure levels are considered adequately known (2–4, 7, 8). This knowledge is scientifically based on the understanding of the specific mechanisms by with EMF interact with biological tissues, such as the induction of electric currents and secondary magnetic fields in the body for low frequency fields and energy deposition with increasing in temperature for high frequency fields. For these effects there are available data on the thresholds above which the effects can appear, and protective Exposure Limit Values for occupational exposures, higher compared with the limits for the general public, are defined. This information is sufficient to perform risk assessment within companies, in order to classify occupationally exposed workers and take the necessary preventive measures (2–4, 7, 8). Workers such as MRI operators, welders, telecommunication antennas' installers and maintainers, power plants operators, employees of radiofrequency-based thermal treatments machineries are usually considered “EMF exposed workers” in the majority of the companies after an OSH risk assessment according to the European legislation, but the problem is that these considerations are not directly applicable to the workers at particular risk. For these workers, and in particular for those with AIMD or AWMD, also EMF exposure levels more similar to the emissions of sources commonly used in all the industrial and office environments could be potentially harmful, if the workers are attached or very close to the sources (e.g., a subject with a pacemaker has to keep the mobile phone about at least 15 cm far from the device to avoid an EMI hazard). These workers should not be considered occupationally exposed to EMF, but they may have a potential EMF-related hazard and the legislation requires to specifically consider them for an appropriate EMF risk assessment at the workplace, to define protections including HS (8).

The problem of an increased susceptibility of some workers to work-related hazards is well known and fundamental for all OSH risk prevention activities, including HS. Nevertheless, for electromagnetic hazards the category of the particularly sensitive workers with AIMD or AWMD is peculiar for various reasons. These devices are “active” meaning that their functioning is based on electronic circuits with or without batteries. Accordingly, the electric, magnetic and electromagnetic fields of the devices can be subjected to potential interference problems when the workers are close to other EMF sources. This can result in temporary, if not permanent, alterations of sensing, functioning, and settings of both implanted and body-worn devices. In case of intense exposures, unwanted stimulations originated from the devices are possible, as well as thermal effects determining heating of the devices and/or of the surrounding tissue, especially if the devices contain metal parts. Such malfunctions may constitute a potential risk for workers' health (9).

The number and type of implanted or body-worn active devices have been progressively increased in recent years due to the development of new technologies and of wider medical indications for their use. Moreover, if we refer to occupational settings, it has to be considered that, as the working population is progressively aging (10), the number of workers with such devices is potentially increasing, and will definitely increase in the next decades. In Italy, according to the Italian Hospital Discharge Database, the number of implanted cardiac pacemakers (PM) raised from 36,823 per year in 2001 up to 49,716 per year in 2017, with an increase of 12,829 implants (+35%) per year. Considering Implanted Cardioverter Defibrillators (ICD), 3,141 devices were implanted in Italian hospitals in 2001, while in 2017 24,255 ICDs were implanted, with an increase of 21,114 (+672%) devices (11).

Another issue to possibly be considered is also related to the new applications and sources of EMF rapidly developed with the advancement in technology (12): potential interference risks for active medical devices need to be re-evaluated in case of relevant EMF emitting new sources installed at the workplaces.

According to all these above-mentioned premises, raising awareness on the various types of active implanted and wearable medical devices available on the market is therefore essential for a proper HS of workers who use these devices and are exposed to EMFs. The identification of workers with a particular susceptibility is one of the fundamental requirements for an adequate prevention of the occupational EMF risk.

## 2 Objectives

The aim of this paper is to estimate the diffusion of workers with AIMD and AWMD and the types of the most frequent devices used by interviewing a sample of OPs in Italy. OPs will be asked to report on the workers they follow within occupational HS programs. This will provide an overview of the dimension of the phenomenon of the particular sensitivity in the context of managing electromagnetic hazards at the workplaces, a fundamental issue to be considered for a proper risk assessment and prevention.

## 3 Methods

### 3.1 Study setting and design

We performed a cross-sectional survey among a sample of Italian OPs registered in the Italian National Registry of Occupational Physicians and active in the Region Emilia Romagna and Toscana (13). We targeted at reaching out at least 10% of all the OPs currently registered in the two regions (total number = 1,155). We contacted the OPs during their participation in training courses for the prevention of occupational radiation exposure risks carried on between November 2023 and June 2024, as well as through direct email contacts. Each OP was asked to respond to an online questionnaire (Appendix 1), based on their experience in the HS of workers, estimating the numbers of subjects followed in the previous 12-months who have AIMD and AWMD, and indicating the most frequent types of devices.

The study is carried out within the project BRIC22-ID36 “Evaluation and management of the risk arising from new sources of exposure to electromagnetic fields for the protection of workers with Active Implantable Medical Devices”, funded by the Italian National Institute for Insurance against Accidents at Work (INAIL).

### 3.2 The questionnaire

Data have been collected through an online survey administered to the OPs through Google forms.

The survey has been specifically designed for the OPs, based on a review of current scientific and gray literature aimed at elaborating a non-comprehensive list of AIMD and AIWD currently used and potentially subject to electromagnetic interference. The list has also been shared within all the multi-disciplinary research teams of the BRIC22-ID36 project in order to check it and identify other potential devices of interest. In any case, the questionnaire also included an open item to report any other AIMD or AWMD found during their HS activities.

The questionnaire is short, in order to provide a good compliance from OPs, and consists of 15 items. The first five items investigate general information and characteristics of the OPs, the working sectors they follow most, the number of workers subjected to HS they visited in the previous 12-months and the specific number of workers visited with an EMF-related occupational risk according to the companies' risk assessment documents. Considering this latter point, as anticipated in the introduction, in Italy HS is a medical practice and it is activated only based on the results of OSH risks assessment. Accordingly, OPs visit the workers and decide on their fitness for work based on the hazards they are exposed to. The lists of workers exposed to specific hazards is communicated by the employers to the OPs, so that every OP knows for the specific company how many workers they visited with exposure to electromagnetic hazards certified in the companies' risk assessment documents.

The other nine items of the questionnaire specifically consider different groups of AIMD and AWMD: (1) cardiac pacemakers (PM); (2) implantable cardioverter-defibrillators (ICD); (3) implanted loop-recorders (ILR); (4) cochlear or brainstem auditory implants; (5) implanted central nervous system (CNS) neurostimulators; (6) implanted peripheric nervous system (PNS) neurostimulators; (7) active wearable prostheses for arms or legs or other wearable aids for motor functions; (8) wearable pumps for the infusion of drugs or hormones; (9) wearable hearing aids. Finally, as anticipated above, a further item asks OPs to report any other AIMD or AWMD found during their medical examination of workers in the previous year.

For each device, the survey includes an explanatory image, and the following question: “In the past 12 months, in the context of all HS medical examinations you performed in your role of companies' OP, how many workers with the following AIMD/AWMD did you visit approximately?”

### 3.3 Data analysis

We calculated the total number of workers reported to be visited under occupational HS programs per year by the OPs who filled the questionnaire, including also the specific subgroups of workers visited with an indication of an EMF risk for their job task based on the companies' risk assessment documents. All the different types of medical examinations included in HS programs have been considered, and in particular the most frequently occurring types of visits are pre-employment examinations, periodic examinations and examinations upon request from the workers due to possible work-related problems. Based on the denominator, i.e., the total number of workers visited, we obtained the percentage frequencies corresponding to the proportion of workers with the active implanted and wearable medical devices, based on the numbers of workers with the devices reported by the OPs.

In case of missing information on the total number of workers visited by a single OP, in order to retrieve the total number for all the OPs, we applied to the missing information the average number of workers visited annually based on the respondents.

The total numbers of workers calculated were used to determine the estimate of the percentages of workers carrying AIMD and AIWD, for the different types considered, in Italian companies and therefore have an idea of the possible risk of interference linked to the presence of particularly sensitive workers to be considered in HS activities and the management of electromagnetic hazards.

## 4 Results

### 4.1 Characteristics of the studied population

A total of 132 OPs responded to the questionnaire. Characteristics of OPs and general information about their practice are shown in Table 1. The majority of the responding OPs are aged 51–60 and 61–70, respectively 24.2% (32 OPs) and 31.8% (42 OPs). Additionally, 53.8% (*n* = 71) of OPs are males, while 46.2% (*n* = 61) are females. The most of OPs, that is 45.45% (60 OPs), reported working for various companies from different sectors, 23.48% (31 OPs) reported working in the healthcare sector, 21.21% (28 OPs) reported working in other sectors, 8.33% (11 OPs) reported working in the tertiary sector and only 1.52% (2 OPs) reported working in the construction sector.

**Table 1:** Information and characteristics of the sample of interviewed Occupational Physicians.

		**Age class % (** ***n*** **)**					**Total**
		**31–40**	**41–50**	**51–60**	**61–70**	>**70**	
Gender	M	3.8% (5)	3.8% (5)	9.8% (13)	22.7% (30)	13.6% (18)	53.8% (71)
	F	6.8% (9)	15.2% (20)	14.4% (19)	9.1% (12)	0.8% (1)	46.2% (61)
Main sector where OP perform the Health Surveillance (HS) activity	Construction	0.8% (1)	0.0% (0)	0.0% (0)	0.8% (1)	0.0% (0)	1.5% (2)
	Healthcare	3.0% (4)	3.8% (5)	7.6% (10)	7.6% (10)	1.5% (2)	23.5% (31)
	Tertiary	0.0% (0)	0.8% (1)	2.3% (3)	3.8% (5)	1.5% (2)	8.3% (11)
	Other sectors	0.8% (1)	3.8% (5)	5.3% (7)	7.6% (10)	3.8% (5)	21.2% (28)
	Not possible to identify a single sector	6.1% (8)	10.6% (14)	9.1% (12)	12.1% (16)	7.6% (10)	45.5% (60)
Mean number of workers followed during HS in the previous year (SD)		1,735 (±1,413.6)	1,688 (±937.6)	1,802 (±1301.7)	1,401 (±887.8)	1,137 (±755.7)	1,550 (±1,067.8)
Mean number of workers exposed to EMFs followed during HS in the previous year (SD)		119 (±103.3)	101 (±168.5)	107 (±142.8)	109 (±233.0)	62 (±48.5)	101 (±169.4)

All OPs visited a total of 204,585 workers (including both workers not exposed to EMFs and those defined as *professionally exposed to EMFs* whose work involves exposure to EMFs according to an evaluation performed based on the EU Directive 2013/35 as defined in the introduction) in the previous 12 months within the HS activities they conducted on behalf of the companies, each OP having visited an average of 1550 ± 1067.8 workers. The OPs in the age range 51–60 are those with the highest average number of workers visited in the previous 12 months, with almost 1,802 workers each. Of the total working population considered, OPs reported on average that 6.53% are exposed to EMF based on the risk assessment documents of the companies, for a total number of workers exposed to this risk of 13,360.

### 4.2 Types and reported frequencies of active implanted or wearable devices among workers undergoing health surveillance examinations

Table 2 shows the types and the occurrence of all the devices specifically considered in the questionnaire, within the two categories of AIMD and AWMD. In particular, the most frequently reported AIMD is PM, with a total of 299 workers with the device visited in the past 12 months, which represents 0.15% of the whole number of workers who underwent HS. The following most frequent AIMD is ICD, with 188 workers reported by OPs, which represent 0.09% of the total considered working population visited during HS in the previous 12 months. Overall, AIMDs related to cardiovascular function were by large the most frequent, reaching 0.28% of the total working population examined, when considering together PM, ICD and loop recorders. The least occurrent AIMDs are implanted neurostimulators both for the PNS and the CNS, found only in 39 workers in total, i.e., 0.02% of the working population visited.

**Table 2:** Types and reported frequencies of active implanted or wearable devices among workers undergoing health surveillance examinations with the interviewed Occupational Physicians.

	**Device**	**Total number of workers with the device visited in the past 12 months**	**% of workers with the device on the total number of workers visited (204,585)**
AIMD	Pacemakers	299	0.15%
	ICD	188	0.09%
	Loop recorders	87	0.04%
	Cochlear implants	126	0.06%
	CNS neurostimulators	24	0.01%
	PNS neurostimulators	15	0.01%
AWMD	Active prostheses	36	0.02%
	Drug/hormone pumps	349	0.17%
	Hearing aids	521	0.25%
	Sensors for continuous glucose monitoring	32	0.02%
Other devices (not specified by the OPs, not clear whether AIMD or AWMD)		25	0.01%

For what concerns AWMD, OPs reported 521 workers wearing hearing aids, with an occurrence of 0.26% among workers visited in the previous 12 months. Wearable pumps for the infusion of drugs or hormones were found in 349 workers, which is 0.17% of the total number of workers visited in the previous 12 months. On the other hand, active prostheses were found only in 36 workers, while wearable sensors for continuous glucose monitoring were found in 32.

In addition, OPs reported 25 other workers with either an AIMD or an AWMD, even if they did not mention the specific type of device found.

Considering this, the total number of workers with AIMD resulted in 739, corresponding to the 0.38% of the working population considered. On the other hand, AWMD were reported for a total of 938 workers, which is the 0.46% of workers visited in the previous 12-months.

## 5 Discussion

The results of our questionnaire-based investigation involved 132 OPs, referring to an overall sample of 204,585 workers subjected to HS by the OPs during the previous year, of which 13,360 (6.53%) represented the “workers professionally exposed” to EMFs according to the risk assessment documents and based on the European Directive 2013/35. The analyzed results indicate, as expected, that the most common AIMDs found by the OPs in workers during their daily practice of HS are related to the support of cardiovascular function, and, according to our data, may involve up to 0.3% of the working population. In fact, 55% of OPs visited workers with PM in the previous year: one OP reported having visited 20 workers with PM, twelve others reported having visited 10–15 workers, while the remaining OPs reported lower numbers. 60% of the OPs visited workers with ICD: only two OPs found ICD in 10 workers and three in 6–7 workers, while the remaining OPs reported lower numbers. The ILR was reported by 40% of OPs, with one practitioner indicating having examined 12 workers with this device and two OPs reported having visited respectively 4 or 5, while the others 3 or less. Considering this, the major potential interference problems in the case of an electromagnetic hazard for workers with AIMD could affect subjects with implants for the cardiovascular function, involving 574 workers (Table 2). This data is in line with the number of medical devices implanted in Italy in 2017, when, according to the Italian Hospital Discharge Database, have been implanted 49,716 PMs and 24,255 ICDs (11).

With regard to other types of AIMD, these resulted much less frequent when compared to those related to the cardiovascular apparatus, so that, even grouping together neurostimulators and cochlear implants, our estimates indicate that < 0.1% of the working population could have these types of implanted devices. In particular, our data show that during HS only the 35% of the OPs reported to have visited workers with cochlear/brainstem hearing implants, the 14.4% examined workers with central neurostimulator and the 10.6% visited workers with peripheric neurostimulators (Table 2).

The most studied AIMDs are PM and ICD, in terms of interference with EMFs (14–20). Although *in vivo* data collected during occupational activities regarding AIMDs and possible interference problems are scarce, they suggest that such issues may occur but are reversible and fortunately clinically silent, and could be discovered during the periodical clinical checks of the devices (5, 17–19). In fact, only interference occurring at the same time as a necessary stimulation could result in severe clinical manifestations, since a modification in the sensing function of a PM may not recognize a cardiac rhythm alteration. Moreover, interference between EMFs and cardiac active devices could also result in an unnecessary stimulation (8, 9).

For what concerns *in vivo* occupational studies, Souques et al. (21) found no interference problems studying an electricity company where three workers with ICD were exposed to 50 Hz EMF. Moreover, Tiikkaja et al. (22) studied 11 volunteer workers with PM and 13 with ICD exposed to 2–200 Hz magnetic fields, electronic article surveillance (EAS) gate emitting EMFs, an induction cooktop and a metal inert gas welding machine, finding no interference problems in any of the scenarios involving PM with bipolar settings and ICDs. They only found interference between three PMs with unipolar settings and 2–200 Hz magnetic fields and one of them showed interference problems with the EAS gate and the welding machine (22).

On the other hand, analyzing studies performed on trunk simulators, Mattei et al. found significant interference problems between AIMD and static magnetic fields of MRI scanners (18–20). Korpinen et al. found interference problems in several types of PM in work scenarios with workers struck by spark discharges or immersed in magnetic fields of shunt reactors at 400 kV substations (16, 17).

For what concerns interference in standard exposure conditions, some studies involving various types of AIMD show that interference problems may occur more often in case of devices with unipolar settings, while electromagnetic interference affects less the most recent devices with bipolar configurations, as depicted by Huang et al. in case of GSM mobile phones exposure (15) or by Guag et al. (14) in case of walking-through metal detector security systems. On the other hand, it should also be considered that AIMD classified as “MRI-conditional”, which can be used while undergoing a MRI scan as patients after an appropriate setting of the devices (23, 24), cannot be evaluated as safe for workers exposed to EMF e.g., as MRI operators, since it is not possible to modify the settings of the devices every time the workers would need to approach closely to the MRI scanner (8, 9).

Regardless the exposure conditions, a specific individual risk assessment needs to be performed for workers with AIMD or AWMD. For AIMD, the European Standards of the family EN 50527 provide guidances to perform such risk assessment. The particular standard EN 50527-2-1:2016 contains specific guidance for pacemakers (25), whereas the EN 50527-2-2:2018 focuses on ICD (26).

No international standards or technical documents are currently available, on the other hand, for the individual risk assessment of workers with AWMDs exposed to EMF (8, 9). Nevertheless, our data show that some of these devices are frequent within the working population, and should deserve considerations during HS activities with respect to possible EMF interference problems. For what concerns AWMD, the most frequent devices were hormone/drug pumps and hearing aids, for a total of 870 devices, constituting ~0.4% of the total workers subjected to HS by OPs in the previous year.

External hearing aids were reported by 93 OPs (70.5%): six doctors reported having visited more than 10 workers with these devices, of which two OPs reported having visited 50 (Table 2). It should be noted that interferences in these cases may be heard as buzzing noises by the subjects (27): in principle it is questionable whether this represents a real direct adverse effect to be considered for prevention, but it should be considered that in many workplaces it is fundamental to be able to hear alarm and warning sirens for the prevention of occupational accidents, avoid any indirect consequence related to a malfunction of the wearable devices. Furthermore, this type of malfunction could lead to a loss of concentration by the worker which could have consequences on safety.

The other frequent AWMD resulted from our investigation are the pumps for the infusion of hormones and/or drugs: 94 OPs (71.2%) visited at least one worker with this device, and in ten cases the number of workers involved was >5 (maximum value = 20) (Table 2). Case reports describing EMI phenomena in wearable infusion pumps at workplaces have been documented in the literature (28, 29). In this case, the effects of the interferences are potentially severe, as they can have an impact on the appropriate delivery of the therapeutic treatments (30).

Consider other types of AWMD, we did not find that active functional limb prosthesis and/or other AWMDs assisting motor functions are common in the Italian working population nowadays: only 17 OPs (12.9%) reported to have visited such workers, for a total of only 36 workers.

Finally, it is interesting the data related to a further type of AWMD, directly reported by the OPs as it was not included as a specific item within the administered questionnaire: doctors estimated a number of 32 workers, presumably affected by diabetes, with wearable active sensors for glucose monitoring visited in the previous year during HS. It should be underlined that this data could be underestimated in our study as we did not specifically ask for this device to the OPs.

Summarizing our estimates of the workers with AIMD and AWMD present at the workplaces, it has to be considered that these workers are definitely at increased risk of EMF-indirect effects and represent about the 1% of the working population. Nevertheless, the data referred by the OPs indicate that ~6.5% of the whole working population they follow within HS programs for the companies are occupationally exposed to EMF based on a risk assessment performed according to the EU Directive 2013/35: if workers with AIMD or AWMD are included in this exposed subgroup, they must be considered as “workers at particular risk” according to the legislation and specific preventive measures need to be taken (4). Another aspect that must be considered is the increasing diffusion of AIMD and AWMD, as well as the aging of the working population and the ubiquity of EMF emitting sources at the workplaces, and the new technologies and sources developed: it is therefore fundamental that OPs know the most common devices that can be affected by interference problems, in order to carry out appropriate HS programs for risk prevention and promotion of workers health and wellbeing.

Despite the new data provided, it should be recognized that the present study has some limitations. First, an important limitation is related to the nature of the study and its cross-sectional design, based on a survey with self-reported data from the OPs. Even if the doctors are experts in their field, misclassification biases related both to the types of devices investigated as well as to the number of workers visited, to the number of workers considered as occupationally exposed to EMF according to the EU legislation and to the number of workers with the devices cannot be excluded. These latter points may deserve further investigations and revisions of the questionnaire used, as it should be relevant to understand how many workers with AIMD or AWMD are estimated to be employed with job tasks exposing them to a professional EMF risk. Finally, considering the representativeness of the sample studied, it should be considered that the OPs surveyed cannot be considered fully representative, being only the 10%, of all the OPs registered in the two Italian regions where the study has been set. Nevertheless, considering the huge number of workers they reported to follow within companies' HS programs, i.e. more than 200,000, distributed across thousands of different companies covering the main occupational sectors, it should be considered that the answers of the investigated sample of OPs can give a full picture of the types of companies and occupational hazards distributed in the two Italian regions where the sample has been collected.

## 6 Conclusions

The results of our study show that the 0.8% of the working population in two Italian regions (i.e., Emilia Romagna and Tuscany) can be estimated as “particularly at risk” in the context of managing electromagnetic hazards at the workplaces for the presence of active implanted or body-worn medical devices. According to our study, the most common AIMD are cardiologic devices (PMs, ICDs, ILRs), while the most common AWMD are hormones/drugs pumps and hearing aids. Very scant data are available on the frequency of neurostimulators, cochlear/brainstem implants and wearable active prostheses among workers. This condition of increased susceptibility must be carefully considered by OPs during HS activities, and it should be specified that potential interference problems could also occur for levels of exposure to EMFs comparable to those measurable for non-professionally exposed populations, in the case of particular proximity to relevant sources. Adequate health surveillance and fitness-for-work evaluation of these workers must be carefully implemented to reduce this risk, also by consulting, in specific situations, the devices' manufacturers and/or the medical specialists in the disease(s) requiring the devices.

The data presented in this work provide a solid foundation for quantifying the actual impact of workers with AIMD and AWMD on the overall framework of HS programs. Additionally, they help better focus efforts on improving awareness and sensitivity among OPs toward this particular group of workers.

## Data Availability

The raw data supporting the conclusions of this article will be made available by the authors, without undue reservation.
