# Supplementary material for: Results of a national survey among occupational physicians to estimate the number of workers with active medical devices and their types in the context of managing electromagnetic hazards
Source: Front Public Health. 2025 Jul 28;13:1599754. doi: 10.3389/fpubh.2025.1599754 (PMC12336113; doi:10.3389/fpubh.2025.1599754)
Supplement: Supplementary file 1 [file Data_Sheet_1.docx]

**Appendix 1 – The questionnaire “Workers with Active Implantable Medical Devices and Active Wearable Medical Devices: a survey for Occupational Health Practitioners”**

# 1a) General information on the responder *Gender*

*Mark only one oval.*


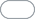
 Male
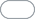
 Female


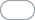
 Prefer not to answer


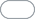
Other:

**1b) General information on the responder** *Age (years)*

*Mark only one oval.*


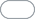
 <30


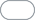
 31-40


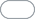
 41-50


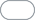
 51-60


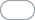
 61-70


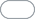
 >70


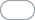
 Prefer not to answer


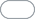
Other: _________________________________________________________________

#

# 2a) General information on the responder's activity.

**For which companies do you mostly work as Occupational Health Practitioner?**

*Mark only one oval.*


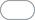
 Agricultural companies
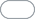
 Construction companies
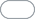
 Industrial companies
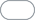
 Healthcare companies


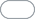
 Other services companies (excluding healthcare)


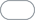
 Not possible to give an univocal answer / working for various companies from different sectors

# 2b) General information on the responder's activity.

Overall, in the companies you follow as OHP, how many workers, approximately, have undergone an Occupational Health Surveillance programme including a medical examination during the last year?

NB: Please write down here below an approximate number. We know this will be probably not equal to the exact number of workers followed, but we hope it would be possible to provide us with a reliable estimate, close to the actual situation. If this would not be possible, please write "don't know"

_______________________________________________________________________

# 2c) General information on the responder's activity.

Overall, during the last year in the companies you follow as OHP, how many workers, approximately, have undergone a specific Occupational Health Surveillance programme, including a medical examination, for an exposure to ElectroMagnetic Fields workplace sources?

NB: Please write down here below an approximate number. We know this will be probably not equal to the exact number of workers followed, but we hope it would be possible to provide us with a reliable estimate, close to the actual situation. If this would not be possible, please write "don't know"

______________________________________________________________________________

# 3) Workers with Active Implantable Medical Devices (AIMD

During last year, among the workers who underwent medical examinations within an occupational health surveillance program in the companies you follow as OHP, how many of them have been found implanted with a cardiac pacemaker?

NB: Please write down here below an approximate number. We know this will be probably not equal to the exact number of workers followed, but we hope it would be possible to provide us with a reliable estimate, close to the actual situation. If this would not be possible, please write "don't know"


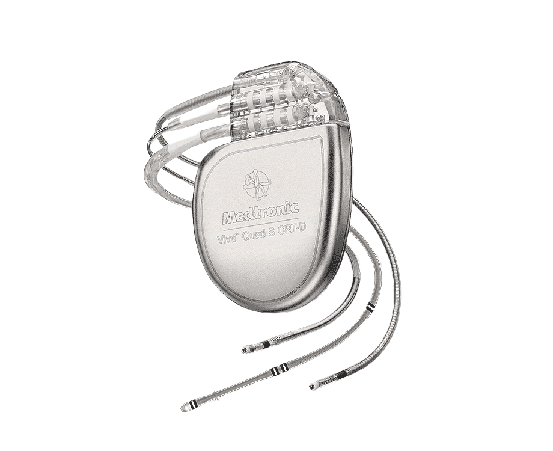
______________________________________________________________________________

# 4) Workers with Active Implantable Medical Devices (AIMD)

During last year, among the workers who underwent medical examinations within an occupational health surveillance program in the companies you follow as OHP, how many of them have been found implanted with a cardiac defibrillator?

NB: Please write down here below an approximate number. We know this will be probably not equal to the exact number of workers followed, but we hope it would be possible to provide us with a reliable estimate, close to the actual situation. If this would not be possible, please write "don't know"


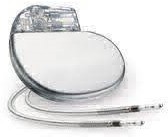
______________________________________________________________________________

# 5) Workers with Active Implantable Medical Devices (AIMD)

During last year, among the workers who underwent medical examinations within an occupational health surveillance program in the companies you follow as OHP, how many of them have been found implanted with a loop recorder?

NB: Please write down here below an approximate number. We know this will be probably not equal to the exact number of workers followed, but we hope it would be possible to provide us with a reliable estimate, close to the actual situation. If this would not be possible, please write "don't know"


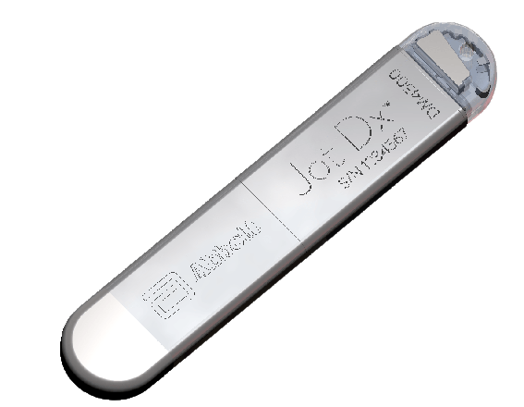
_____________________________________________________________________________

# 6) Workers with Active Implantable Medical Devices (AIMD)

During last year, among the workers who underwent medical examinations within an occupational health surveillance program in the companies you follow as OHP, how many of them have been found implanted with a cochlear implant or an auditory brainstem implant?

NB: Please write down here below an approximate number. We know this will be probably not equal to the exact number of workers followed, but we hope it would be possible to provide us with a reliable estimate, close to the actual situation. If this would not be possible, please write "don't know"


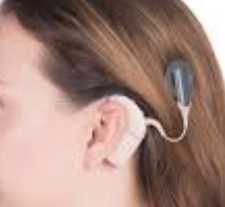
______________________________________________________________________________

# 7) Workers with Active Implantable Medical Devices (AIMD)

During last year, among the workers who underwent medical examinations within an occupational health surveillance program in the companies you follow as OHP, how many of them have been found implanted with a central nervous system stimulator (e.g. Deep Brain Stimulator, subdural spinal neurostimulator)?

NB: Please write down here below an approximate number. We know this will be probably not equal to the exact number of workers followed, but we hope it would be possible to provide us with a reliable estimate, close to the actual situation. If this would not be possible, please write "don't know"


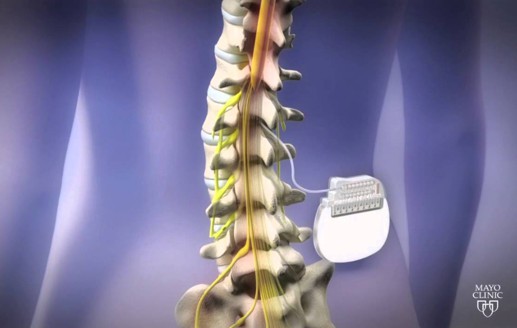
______________________________________________________________________________

# 8) Workers with Active Implantable Medical Devices (AIMD)

During last year, among the workers who underwent medical examinations within an occupational health surveillance program in the companies you follow as OHP, how many of them have been found implanted with a peripheral nerve stimulator?

NB: Please write down here below an approximate number. We know this will be probably not equal to the exact number of workers followed, but we hope it would be possible to provide us with a reliable estimate, close to the actual situation. If this would not be possible, please write "don't know"


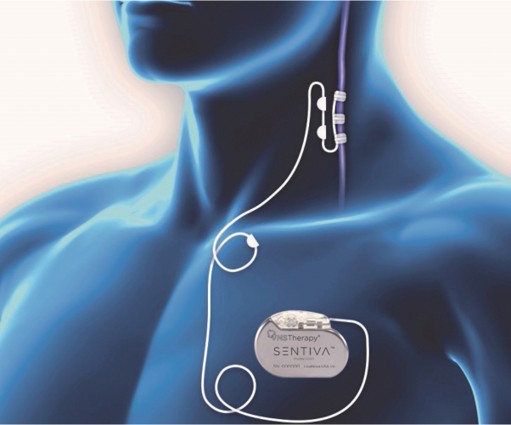
______________________________________________________________________________

# 9) Workers with Active Wearable Medical Devices (AWMD)

During last year, among the workers who underwent medical examinations within an occupational health surveillance program in the companies you follow as OHP, how many of them have been found wearing a functional active prothesis or another wearable auxiliary device for motor functions?

NB: Please write down here below an approximate number. We know this will be probably not equal to the exact number of workers followed, but we hope it would be possible to provide us with a reliable estimate, close to the actual situation. If this would not be possible, please write "don't know"


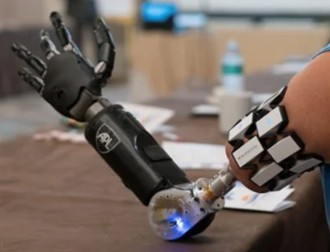
______________________________________________________________________

# 10) Workers with Active Wearable Medical Devices (AWMD)

During last year, among the workers who underwent medical examinations within an occupational health surveillance program in the companies you follow as OHP, how many of them have been found wearing a drugs/hormones infusion pump?

NB: Please write down here below an approximate number. We know this will be probably not equal to the exact number of workers followed, but we hope it would be possible to provide us with a reliable estimate, close to the actual situation. If this would not be possible, please write "don't know"


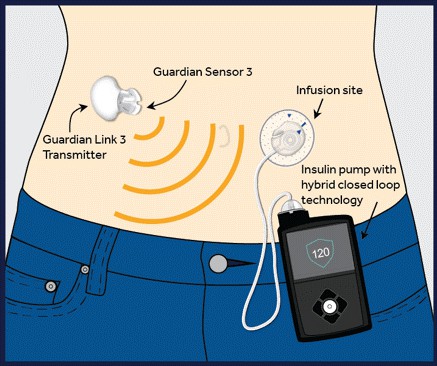
_____________________________________________________________________

# 11) Workers with Active Wearable Medical Devices (AWMD)

During last year, among the workers who underwent medical examinations within an occupational health surveillance program in the companies you follow as OHP, how many of them have been found wearing a hearing aid?

NB: Please write down here below an approximate number. We know this will be probably not equal to the exact number of workers followed, but we hope it would be possible to provide us with a reliable estimate, close to the actual situation. If this would not be possible, please write "don't know"

______________________________________________________________________________


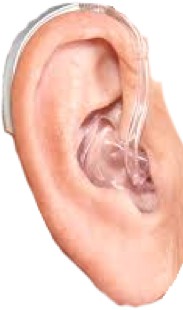


# 12) Workers with Active Wearable Medical Devices (AWMD)

During last year, among the workers who underwent medical examinations within an occupational health surveillance program in the companies you follow as OHP, how many of them have been found with other type(s) of AIMD or AWMD? Which device, in case?

NB: Please write down here below an approximate number. We know this will be probably not equal to the exact number of workers followed, but we hope it would be possible to provide us with a reliable estimate, close to the actual situation. If you have been informed of workers with other type(s) of AIMD or AWMD not listed above, please write down the name of the device(s) just after the number reported. If you haven't been informed of workers with other type(s) of AIMD or AWMD not listed above, please write down "0". If it is not possible to give an answer to this item, please write "don't know” ______________________________________________________________________________
